# Prevalence of cutaneous adverse events associated with long-term disease-modifying therapy and their impact on health-related quality of life in patients with multiple sclerosis: a cross-sectional study

**DOI:** 10.1186/1471-2377-13-146

**Published:** 2013-10-16

**Authors:** Deepak MW Balak, Gerald JD Hengstman, Enes Hajdarbegovic, Rob JP van den Brule, Raymond MM Hupperts, Hok Bing Thio

**Affiliations:** 1 Department of Dermatology, Erasmus Medical Center, Burg. s’ Jacobsplein 51, 3015 CA, Rotterdam, the Netherlands; 2 Department of Neurology, Catharina Ziekenhuis, Regional MS Centrum Oost-Brabant, Eindhoven, the Netherlands; 3 Biogen Idec International B.V., Badhoevedorp, the Netherlands; 4 Department of Neurology, Academic MS Centre Limburg, Sittard-Geleen, the Netherlands

**Keywords:** Skin reactions, MS, Quality of life, Glatiramer acetate, Interferon-beta, Injections

## Abstract

**Background:**

Glatiramer acetate (GA) and interferon-beta (IFN-β) are disease-modifying therapies (DMTs) for multiple sclerosis that are administered through subcutaneous (SC) or intramuscular (IM) injections. Skin reactions associated with DMTs are common and may influence patient’s health-related quality of life (QoL). We aimed to determine the prevalence of cutaneous adverse events associated with long-term DMT use, and to assess the impact of cutaneous adverse events on QoL.

**Methods:**

A cross-sectional study among patients with multiple sclerosis who had been treated with their first DMT for at least 2 years. Cutaneous events were assessed from photographs of injection-sites by dermatologists blinded for DMT. Generic and dermatology-specific health-related QoL were assessed using validated patient-reported questionnaires.

**Results:**

A total of 229 patients were enrolled, of whom 156 (68%) had at least one skin reaction. The prevalence of cutaneous adverse events was higher for SC DMTs (75-82%) compared to IM DMT (41%) (P < 0.001). Erythema and lipoatrophy were the most common skin reactions, observed in 156 (68%) and 45 (20%) patients, respectively. Dermatology-specific, but not generic, QoL was significantly lower among patients with skin reactions compared to those without.

**Conclusions:**

The prevalence of cutaneous adverse events was high in long-term DMT-treatment. Patients with cutaneous adverse events had a lower perceived dermatology-specific QoL.

## Background

Multiple sclerosis (MS) is an immune-mediated inflammatory disease of the central nervous system that is characterized by inflammation, demyelination, and neurodegeneration within the brain and the spinal cord [1]. Most cases present as relapsing-remitting MS that eventually progresses to a secondary progressive stage [2]. Treatment of MS is currently limited to reducing disease progression via disease-modifying treatments (DMTs) that modulate the immune system [3]. Approved first-line DMTs are glatiramer acetate (GA) and interferon-β (IFN-β), which require self-administration via subcutaneous (SC) or intramuscular (IM) injections. To date, four first-line injectable DMTs are approved for the treatment of MS: SC GA (Copaxone), which is injected once daily; SC IFN-β-1b (Betaferon/Betaseron) injected once every other day; SC IFN-β-1a (Rebif) injected three times a week; and IM IFN-β-1a (Avonex) injected once a week.

GA and IFN-β have been used for more than a decade, and their efficacy and safety-profile are well documented [3]. Skin reactions appear to be frequently occurring adverse events during DMT treatment. A systematic review from 2012 reported relatively high prevalences of cutaneous adverse events associated with DMT, with up to 90% for patients treated with a SC DMT and up to 33% among those treated with an IM DMT [4]. The majority of the reported cutaneous adverse events comprise local injection-site reactions such as erythema, swelling, and induration of the skin surrounding the injection-site. These skin reactions are usually mild and do not necessitate discontinuation of treatment. Other cutaneous adverse events may be more severe or persisting, such as lipoatrophy, cutaneous necrosis and ulcers, and inflammatory skin diseases like psoriasis and vasculitis [4].

Despite the high frequency of cutaneous adverse events associated with DMT, relatively little is known about their impact of patient’s quality of life during long-term treatment.

In this cross-sectional study, we aimed to determine the prevalence of cutaneous adverse events associated with long-term treatment with DMT in daily clinical practice, to assess the effects of cutaneous adverse events on patient’s quality of life, and to identify factors associated with the occurrence of skin reactions.

## Methods

### Study design

The Dermatological injection site reactions in Multiple Sclerosis (derMiS) study was a cross-sectional study conducted among 15 neurology outpatient clinics in the Netherlands. The study was conducted in the period December 2010 to September 2011. The local medical ethical review committees of the Catharina Ziekenhuis (Eindhoven, the Netherlands) and the Central Committee on Research involving Human Subjects (The Hague, the Netherlands) approved the study protocol. All patients gave written informed consent.

### Study population

Inclusion criteria allowed inclusion of patients who were diagnosed with relapsing-remitting MS or clinically isolated syndrome suggestive of MS and who were treated with their first DMT for 2 years or longer. The DMT considered in this study were SC GA, SC IFN-β-1a, IM IFN-β-1a, and SC IFN-β-1b. Patients were excluded if they were treated with their first DMT for less than 2 years, or if they had switched previously from another DMT. In order to prevent selection bias, all consecutive patients willing to participate were included, irrespective of the presence of skin reactions at the injection site.

### Data collection

All data were collected at a single study-visit. Clinical data that were collected were age, sex, weight, length, atopy status, year of onset of MS, type of DMT, treatment duration of DMT, injection-site locations, and concomitant injectable treatments for diseases other than MS. The occurrence of skin reactions as assessed by the treating neurologist and by patients themselves was recorded.

Further, digital photographs were taken of all injection-sites of each patient, with both a close-up and an overview photograph of each injection-site. All digital photographs were centrally assessed for the occurrence and extent of cutaneous adverse events by dermatologists, who were blinded with respect to the DMT treatment-arm. Cutaneous adverse events were defined as any skin abnormality that was assessed on the digital photographs. Several cutaneous adverse events were predefined prior to assessing the photographs, including erythema, ecchymosis, lipoatrophy, panniculitis, skin necrosis, and skin ulcer.

Patients’ attitudes towards injection-site reactions were recorded using a patient-reported questionnaire. Quality of life was assessed using validated patient-reported questionnaires. Generic health-related quality of life (QoL) was measured using the European Quality of life questionnaire 5 Dimensions (EQ-5D) questionnaire [5]. The EQ-5D uses two scoring systems, with higher scores indicating better QoL. The EQ-5D descriptive system evaluates 5 dimensions (mobility, self-care, usual activities, pain/discomfort and anxiety/depression) in a utility-index of 0 to 1.0. The EQ-5D visual analogue scale (VAS) records patient’s self-rated health on a scale of 0 to 100. Dermatological-specific health-related QoL was measured using the Skindex-17 questionnaire [6]. The Skindex-17 consists of two domains: a psychosocial domain on a scale from 0 to 24, and a symptoms domain on a scale from 0 to 10. Higher Skindex-17 scores indicate lower QoL.

### Data analysis

Data were analysed using descriptive statistical methods. Data are presented as means and standard deviation (SD), or as medians and interquartile range (IQR) when not normally distributed. Differences in categorical variables and continuous variables between the groups were tested with the Kruskal Wallis test or the Chi square test, respectively. We did not correct for multiple testing, considering that valid associations would be missed otherwise [7]. The associations between putative determinants and the occurrence of cutaneous adverse events were assessed by calculating odds ratio (OR) and 95% confidence intervals (CI) using logistic regression. A P-value of 0.05 or less was considered statistically significant.

## Results

### Study population

A total of 229 patients were enrolled. Patient characteristics are presented in Table 1. The majority (73%) of the patients were female, and the median age at time of enrolment was 48 years (IQR 41–54 years). Median duration of MS was 9 years (IQR 4–16 years), and median duration of the DMT-treatment was 5 years (IQR 3–9 years). Forty-four (19%) patients were treated with GA, 60 (26%) with IFN-β-1b, 66 (29%) with SC IFN-β-1a, and 59 (26%) with IM IFN-β-1a. Age of time of enrolment and use of an auto-injector for DMT-administration differed between the four DMT-groups. There were no statistically significant differences in the other demographic or clinical variables.

**Table 1:** Patient characteristics

	**Disease-modifying therapy for MS**					
	**GA**	**SC IFN-β-1b**	**SC IFN-β-1a**	**IM IFN-β-1a**	**Total group**	P-value*
No. of patients	44 (19%)	60 (26%)	66 (29%)	59 (26%)	229 (100%)	
Age in years at time of enrolment	48 (40–54)	51 (44–58)	45 (40–52)	49 (41–55)	48 (41–54)	0.025
No. of female patients	37 (84%)	40 (67%)	46 (70%)	45 (76%)	168 (73%)	0.197
Body mass index in kg/m^2^	25 (22–27)	25 (23–27)	25 (23–27)	25 (22–27)	25 (23–27)	0.871
Duration of MS in years	8 (4–17)	11 (4–17)	7 (3–13)	10 (4–14)	9 (4–16)	0.192
Duration of DMT in years	5 (3–8)	5 (3–12)	4 (3–8)	5 (4–10)	5 (3–9)	0.177
Use of auto-injector for injections of DMT	33 (75%)	52 (87%)	49 (74%)	18 (31%)	152 (66%)	< 0.001

Data are presented as medians (interquartile range) or numbers (percentages).

*Abbreviations*: *GA* glatiramer acetate, *IFN* interferon, *IM* intramuscular, *MS* multiple sclerosis, *SC* subcutaneous.

*Differences between the groups were tested with the Kruskal Wallis test or the Chi square test.

### Prevalence of cutaneous adverse events

**Figure 1:**
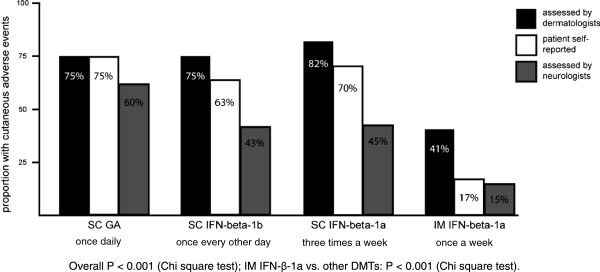
Prevalence of cutaneous adverse events per DMT as reported by dermatologists (black bars), by patients themselves (white bars), and by the treating neurologists (grey bars).

Of all 229 patients, 156 (68%) had at least one cutaneous adverse event as assessed on the digital photographs by the dermatologists (Figure 1). Patients treated with once weekly IM IFN-β-1a had the lowest frequency of cutaneous adverse events, with skin reactions found in 24 (41%) of 59 patients (P < 0.001 vs. other frequently administrated DMTs). The prevalence of cutaneous adverse events in the SC DMT-groups was comparable, ranging from 75% for GA and for SC IFN-β-1b to 82% for SC IFN-β-1a (P = 0.624). The prevalence of cutaneous adverse events in the SC IFN-β-1b group was not statistically different from those treated with SC IFN-β-1a (P = 0.352).

The prevalence of patient self-reported cutaneous adverse events was comparable to that as assessed by the dermatologists (Figure 1). Only in the group of IM IFN-β-1a, the prevalence of skin reactions as self-reported by patients was smaller compared to the prevalence of skin reactions as assessed by the dermatologists. The prevalences of skin reactions reported by the treating neurologists were consistently lower compared to the dermatologists- and patient-reported prevalences.

### Type of cutaneous adverse events

**Figure 2:**
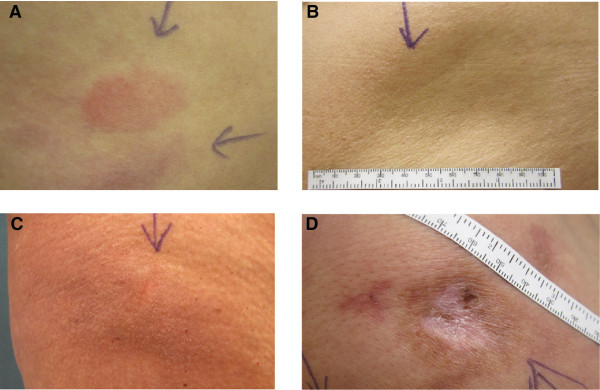
Examples of different types of cutaneous adverse events: erythema (panel A), lipoatrophy (panel B), eczema-like reaction (panel C), and postinflammatory hyperpigmentation and healed scar (panel D).

Overall, erythema and lipoatrophy were the most common skin reactions, which were observed in 156 (68%) and 45 (20%) patients, respectively (Table 2). Less frequently occurring cutaneous reactions were postinflammatory hyperpigmentation (7%), eczema-like reactions (6%), healed scars (3%), urticaria (2%), skin necrosis (0.4%), varicose veins (0.4%), and subcutaneous swelling (0.4%). Figure 2 shows examples of erythema, eczema-like reaction, lipoatrophy, and a healed scar with postinflammatory hyperpigmentation.

**Table 2:** Frequencies of different types of cutaneous adverse events per DMT

	**Disease-modifying therapy for MS**				
	**GA (n = 44)**	**SC IFN-β-1b (n = 60)**	**SC IFN-β-1a (n = 66)**	**IM IFN-β-1a (n = 59)**	**Total group (n = 229)**
Erythema	20 (46%)	39 (65%)	52 (79%)	11 (19%)	156 (68%)
Lipoatrophy	15 (34%)	10 (17%)	11 (17%)	9 (15%)	45 (20%)
Ecchymosis	4 (9%)	7 (12%)	6 (9%)	4 (7%)	21 (9%)
Postinflammatory hyperpigmentation	5 (11%)	7 (11%)	3 (5%)	0 (0%)	15 (7%)
Eczema	1 (2%)	8 (13%)	4 (6%)	1 (2%)	14 (6%)
Healed ulcers	5 (11%)	0 (0%)	2 (3%)	0 (0%)	7 (3%)
Urticaria	0 (0%)	3 (5%)	1 (2%)	0 (0%)	4 (2%)
Necrosis	0 (0%)	1 (2%)	0 (0%)	0 (0%)	1 (0.4%)
Varicose veins	0 (0%)	0 (0%)	0 (0%)	1 (2%)	1 (0.4%)
Subcutaneous swelling	0 (0%)	0 (0%)	0 (0%)	1 (2%)	1 (0.4%)

*Abbreviations*: *DMT* disease-modifying treatment, *GA* glatiramer acetate, *IFN* interferon, *IM* intramuscular, *MS* multiple sclerosis, *SC* subcutaneous.

Erythema was the most common skin reaction in all four DMT-groups. The group treated with IM IFN-β-1a had a lower proportion of erythema (19%) in comparison to the other three DMTs (46% to 79%) (P < 0.001, IM IFN-β-1a vs. the other DMTs). The second-most reported skin reaction was lipoatrophy, which was reported mostly in patients treated with GA. Fifteen (34%) of 44 patients on SC GA were diagnosed with lipoatrophy, which was significantly higher compared to the proportion of lipoatrophy among patients treated with SC IFN-β-1b (17%), SC IFN-β-1a (17%), and IM IFN-β-1a (15%) (P = 0.007, GA vs. the other three DMTs). Frequencies of ecchymosis were roughly comparable between the 4 DMTs. By contrast, postinflammatory hyperpigmentation was found predominantly in patients treated with SC IFN-β-1b (11%) and GA (11%), compared to patients on SC IFN-β-1a (5%) and on IM IFN-β-1a (0%) (P = 0.02, IM IFN-β-1a vs. the other 3 DMTs). The proportion of eczema was higher in patients treated with SC IFN-β-1b (13%), compared to those treated with SC IFN-β-1b (6%), IM IFN-β-1a (2%), and GA (2%) (P = 0.011, SC IFN-β-1b vs. the other DMTs). Cutaneous ulcers were predominantly described in patients treated with GA (11%), whereas the prevalence was lower among patients treated with SC IFN-β-1a (2%), SC IFN-β-1b (0%), and IM IFN-β-1a (0%) (P = 0.003, GA vs. other DMTs). The other cutaneous adverse events occurred too infrequently to assess differential prevalences between the four DMTs.

### Impact on health-related quality of life

Generic QoL as measured by EQ-5D did not differ substantially between patients with and without skin reactions (Table 3). Both the EQ-5D utility score and the EQ-5D VAS were comparable between patients with cutaneous adverse events and patients without cutaneous adverse events.

**Table 3:** Self-reported generic (EQ-5D) and dermatology-specific (Skindex-17) health-related quality of life in patients with and without cutaneous adverse events associated with DMT

	**Cutaneous adverse events**		
	**Yes (n = 156)**	**No (n = 73)**	P value*
***Generic quality of life***			
EQ-5D utility			
median (IQR)	0.727 (0.620–0.848)	0.718 (0.664–0.814)	0.779
mean (SD)	0.725 (0.205)	0.714 (0.218)	
EQ-5D VAS			
median (IQR)	70.0 (60.0–80.0)	75.0 (60.0–80.5)	0.361
mean (SD)	67.3 (20.9)	71.7 (15.8)	
***Dermatology-specific quality of life***			
Skindex-17 Psychosocial			
median (IQR)	1 (0–3)	0 (0–1)	P < 0.001
mean (SD)	2.2 (3.0)	1.0 (2.2)	
Skindex-17 Symptoms			
median (IQR)	2 (1–3)	1 (0–2)	0.002
mean (SD)	2.1 (1.7)	1.5 (1.5)	

*Differences between patients with and without cutaneous adverse events were tested with the Chi square test.

In contrast, dermatology-specific QoL was significantly lower among patients with cutaneous adverse events. The mean (SD) Skindex-17 psychological score was 2.2 (3.0) among the patients with cutaneous adverse events, which was significantly higher in comparison to the group without cutaneous adverse events (mean score 1.0 (SD 2.2)) (P < 0.001). Likewise, the mean (SD) Skindex-17 symptom score was relatively high in the group with cutaneous adverse events than compared to patients without cutaneous adverse events (2.1 (1.7) vs. 1.5 (1.5); P = 0.002).

Although patients with cutaneous adverse events had significantly higher Skindex-17 psychological and symptoms scores than did patients without cutaneous adverse events, the proportion of patients with a severe impairment of QoL was relatively small. Only 10 (4%) patients had high Skindex-17 psychosocial score indicating a severe impairment of their QoL. Eighteen (8%) patients had high Skindex-17 symptoms scores indicating a large impact on their QoL.

In a subgroup analysis we did not find any association between specific cutaneous adverse events and lower QoL (data not shown).

### Patients’ attitudes towards cutaneous adverse events

A majority (58%) of all 299 patients felt it was important that their MS treatment did not lead to cutaneous adverse events, whereas 73 (32%) patients felt it was of little importance, and 23 (10%) patients felt it was not important. Of the 126 patients who reported to have cutaneous adverse events to their DMT, 24 (19%) patients indicated the cutaneous reactions to be very cosmetically disfiguring, 29 (23%) to be disfiguring, and 52 (41%) and 21 (17%) indicated the cutaneous reactions to be a little disfiguring or not disfiguring at all, respectively. Most patients (77%) reported that cutaneous reactions did not influence their choice of clothing, while 15 (12%) and 14 (11%) patients reported a large to a very large influence of cutaneous reactions on their choice of clothing, respectively. Twenty-four (19%) patients indicated that cutaneous reactions greatly impacted their overall contentment with the DMT, 41 (33%) indicated a little impact, and 61 (48%) indicated that cutaneous reactions did not impact their overall contentment of their DMT. Finally, 9 (4%) patients reported to have missed injections of their DMT in the past because of cutaneous adverse events.

### Factors associated with cutaneous adverse events

We assessed the associations between the occurrence of local injection-site reactions and lipoatrophy with four putative determinants: atopy, use of auto-injector, sex, and body mass index. None of these 4 factors were statistically significantly associated with the occurrence of cutaneous adverse events (Table 4). There was a trend that female sex was associated with the occurrence of lipoatrophy, however, this association was not statistically significant.

**Table 4:** Putative factors associated with local injection-site reactions and lipoatrophy

	Local injection-site reactions*			**Lipoatrophy**		
	**Odds ratio**	**95% CI**	**P-value**	**Odds ratio**	**95% CI**	**P-value**
Atopy	1.14	0.59–2.21	0.692	1.17	0.55–2.49	0.691
Use of auto-injector	1.45	0.80–2.64	0.219	1.01	0.49–2.07	0.977
Sex	0.88	0.46–1.68	0.690	0.41	0.16–1.03	0.058
Body mass index	1.00	0.94–1.07	0.898	0.98	0.91–1.06	0.629

*Local injections-site reactions include erythema, ecchymosis, and subcutaneous swelling.

## Discussion

In this cross-sectional study among 229 MS patients treated with their first DMT for at least 2 years, 156 (68%) patients were diagnosed with a cutaneous adverse event, with erythema and lipoatrophy being the most common occurring cutaneous events. Patients with a cutaneous adverse event appeared to have a lower dermatology-specific, but not generic, health-related quality of life as compared to those without a cutaneous adverse event.

To appreciate these findings, several limitations need to be considered. First, we included patients who were treated with their first DMT continuously for a treatment period of at least 2 years. Patients who experienced severe cutaneous adverse events to their DMT or patients who experienced cutaneous adverse events as bothersome are likely to have discontinued their treatment and switched to another DMT before 2 years of treatment. Second, our study is based on cross-sectional data. Considering that the frequency of administration differs between the 4 DMTs, the time periods between the moment of last DMT-injection and the moment of the photograph will be different for the 4 DMTs. This may affect the assessment of in particular passing and transient skin reactions such as erythema. Consequently, this may introduce a bias, leading to either under- or over-reporting of cutaneous adverse events. Finally, we assessed skin reactions from digital 2-D photographs. Considering that we did not have 3-D photographs available, there may have been underreporting of mild cases of lipoatrophy presenting with subtle skin depressions. The main strengths of this study are its large sample size, that skin reactions associated with DMTs were the primary outcome, and that the assessment of the skin reactions were done by dermatologists blinded for the treatment. Furthermore, we only included patients who used one type of DMT making it possible to causally link type of DMT to the adverse skin reactions.

Our results demonstrate that in daily clinical practice long-term DMT-use is associated with a high frequency of cutaneous adverse events up to 68% of patients. These results are in line with previous studies, in which the frequency of skin reactions to DMT seem equally high. In an observational prospective multicentre study from Switzerland, 214 (52%) of 412 MS patients had a skin reaction to their DMT [8]. Furthermore, after a one-year follow up period 10% of these patients had developed lipoatrophy, while 3% had developed skin necrosis.

Local injection-site reactions as erythema appear to be dependent on the mode of administration, because the subcutaneous DMTs are in particular associated with erythema, while patients treated with once weekly intramuscular DMT had the lowest prevalence of erythema. By contrast, the occurrence of lipoatrophy seems to be associated with the DMT rather than with mode of administration. GA was associated with the highest prevalence of lipoatrophy, while the three IFN-formulations had relatively few reports of lipoatrophy. This observation is in line with previous reported cases of lipoatrophy with DMT-use, in which most cases involved patients treated with GA [4]. However, an unexpected finding was lipoatrophy occurring in 15% of patients on IM IFN-β-1a. The occurrence of lipoatrophy in patients on IM IFN-β-1a may be explained by an incorrect injection-site technique with subcutaneous instead of intramuscular administration [9].

In this study, generic health-related quality of life as measured by EQ-5D was not affected by the occurrence of skin reactions. By contrast, dermatology-specific health-related quality of life was substantial lower among patients who were diagnosed with cutaneous adverse events. However, the proportion with severe impairment was relatively low, even for patients with lipoatrophy, which in general is an irreversible and often cosmetic disfiguring cutaneous adverse event. This low impact on health-related quality of life may be explained by the design and the inclusion-criteria of the study. Patients with cutaneous adverse events resulting in a reduced quality of life may have stopped their specific treatment within the first two years of treatment, thus making them not eligible to participate in this study.

Several studies have shown that skin reactions are associated with a reduced treatment adherence to DMT. Severe and persisting skin reactions may lead to loss of quality of life, which may also lead to reduced adherence to DMT [10,11]. Previous studies have shown that a reduced treatment adherence to DMT is associated with suboptimal treatment outcomes [12,13], making skin reactions associated with DMTs clinically relevant for the treating neurologists. In a retrospective study among 643 MS patients, skin reactions were a reason for non-adherence to their DMT in 9% of all patients [14]. In an observational prospective study among 414 MS patients treated with a DMT for at least 2 years, more than half (52%) of all patients were diagnosed with a skin reaction, while patients reported injection-site reactions as the most common reason for wanting treatment discontinuation [8]. In our study, a small proportion (4%) of patients reported to have missed injections because of skin reactions. This may be explained by the low impact of cutaneous adverse events on health-related quality of life in the studied population.

An interesting finding in this study is that the treating neurologists reported consistently lower prevalences of cutaneous adverse events compared to the dermatologists, emphasising that skin reactions seem to be underrecognized by neurologists. Treating neurologists should probably question patients more carefully for cutaneous adverse events and pay attention to these specific side effects during their physical examination.

## Conclusions

In conclusion, our results show that cutaneous adverse events are common with long-term DMT treatment, that cutaneous reactions mostly involve local injection-site reactions and lipoatrophy, and that cutaneous adverse events somewhat lowers dermatologic-specific health-related quality of life of patients with MS who have been treated for over two years with an injectable DMT.

## Competing interests

DB declared no conflict of interest. GH has received consulting fees and lecture fees from Biogen Idec, Merck-Serono, Novartis, Teva and Sanofi-Aventis. EH declared no conflict of interest. RB is employed as Medical Science Liaison at Biogen Idec. RH received honoraria for lectures, advisory boards, expert meetings and unrestricted grants from Biogen-Idec, Merck-Serono, Teva, Novartis and Bayer. HT received research, speaking and consulting support from Biogen Idec and Teva.

## Authors’ contributions

DB participated in the acquisition of data, carried out the analyses, and wrote the manuscript. GH participated in the design of the study and helped to draft the manuscript. EH designed the study, participated in the analyses, and helped to draft the manuscript. RB coordinated the study and helped to draft the manuscript. RH participated in data collection and helped to draft the manuscript. HT conceived the study and participated in the design. All authors read and approved the final manuscript.

## Pre-publication history

The pre-publication history for this paper can be accessed here:

http://www.biomedcentral.com/1471-2377/13/146/prepub
